# Supplementary material for: USP30 inhibition induces mitophagy and reduces oxidative stress in parkin-deficient human neurons
Source: Cell Death Dis. 2024 Jan 15;15(1):52. doi: 10.1038/s41419-024-06439-6 (PMC10789816; doi:10.1038/s41419-024-06439-6)
Supplement: Supplementary file 1 — Supplemental Material [file 41419_2024_6439_MOESM1_ESM.pdf]

Supplementary Materials

USP30 inhibition induces mitophagy and reduces oxidative stress in parkin-deficient human neurons

Justyna Okarmus[1], Jette Bach Agergaard[1], Tina C. Stummann[2], Henriette Haukedal[3], Malene Ambjørn[2], Kristine K. Freude[3], Karina Fog[2], Morten Meyer[1,4,5]

[1]Department of Neurobiology Research, Institute of Molecular Medicine, University of Southern Denmark, J.B. Winsløws Vej 21, 5000 Odense C, Denmark; [2]Neuroscience, H. Lundbeck A/S, Ottiliavej 9, 2500 Valby, Denmark; [3]Department of Veterinary and Animal Sciences, Faculty of Health and Medical Sciences, University of Copenhagen, Grønnegaardsvej 7, 1870 Frederiksberg C, Denmark; [4]Department of Neurology, Odense University Hospital, J.B. Winsløws Vej 4, 5000 Odense C, Denmark; [5]BRIDGE - Brain Research Inter-Disciplinary Guided Excellence, Department of Clinical Research, University of Southern Denmark, J.B. Winsløws Vej 19, 5000 Odense C, Denmark

Corresponding author:

Morten Meyer, Ph.D.
Department of Neurobiology Research
Institute of Molecular Medicine
University of Southern Denmark
J.B. Winsløws Vej 21, st.
DK-5000 Odense C, Denmark
Telephone: +45 65503802
E-mail: mmeyer@health.sdu.dk

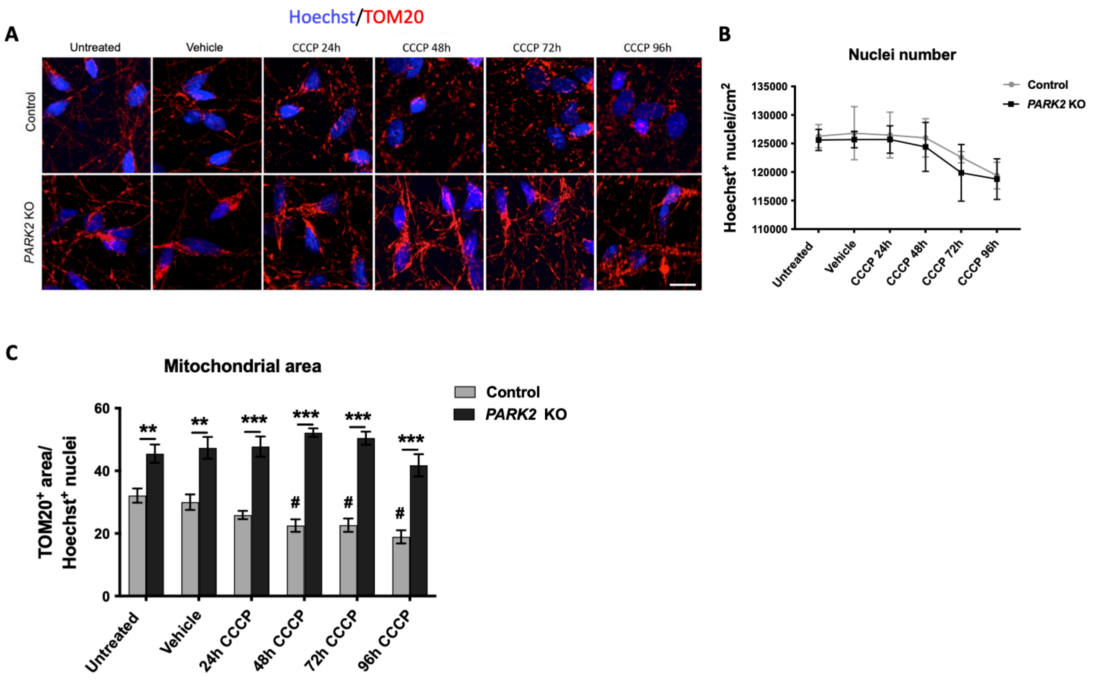

Figure S1: Impaired CCCP-induced TOM20 degradation in *PARK2* KO neurons

A) *PARK2* KO and control neurons were untreated or treated with vehicle (DMSO) or 10 µM CCCP for 24, 48, 72, and 96 hrs and immunofluorescence stained for TOM20 (red) and Hoechst (blue) to visualize mitochondria and nuclei as indicated. Scale bar: 10 µm. **B)** No significant differences in total cell numbers were observed upon CCCP or vehicle (0.1% DMSO) exposure although there was a clear trend towards fewer cells after 72 and 96 hrs. **C)** Quantification of TOM20+ mitochondrial area normalized to number of Hoechst+ nuclei showed that the mitochondrial area in control neurons was significantly reduced after 48, 72, and 96 hrs of CCCP treatment. In contrast, mitochondria were retained in the *PARK2* KO neurons. Vehicle (0.1% DMSO) did not affect area of TOM20 immunoreactivity. Data presented as mean±SEM, n=9 technical replicates, data from 3 independent differentiations, Significant differences are indicated by $**p < 0.01$, $***p < 0.001$ (control vs. *PARK2* KO), $^{\#}p < 0.05$ (untreated control vs. CCCP-treated control), one-way ANOVA followed by Dunnett's post hoc test for multiple comparisons

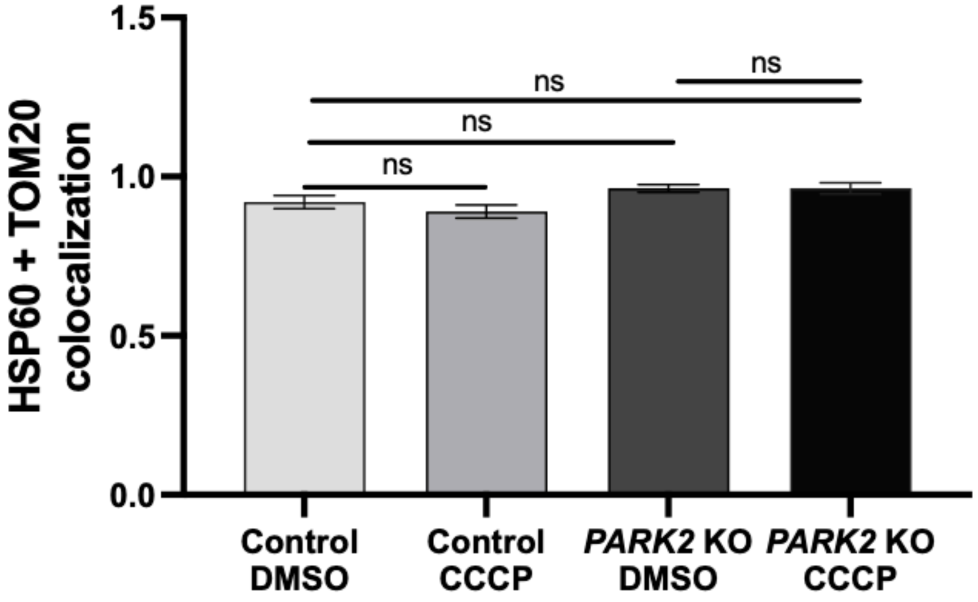

Figure S2: Colocalization analysis of mitochondrial markers (HSP60 and TOM20).

Immunocytochemical colocalization analysis of HSP60 and TOM20 (data presented in *Figs. 2AB*) revealed no significant difference between groups (control DMSO: 88-95%; control CCCP: 87-92%; *PARK2* KO DMSO: 94-98%; *PARK2* KO CCCP: 93-99%). Data presented as mean±SEM, n=9 technical replicates, data from 3 independent differentiations. ns: non significant, one-way ANOVA followed by Dunnett's post hoc test for multiple comparisons.

Figure S3

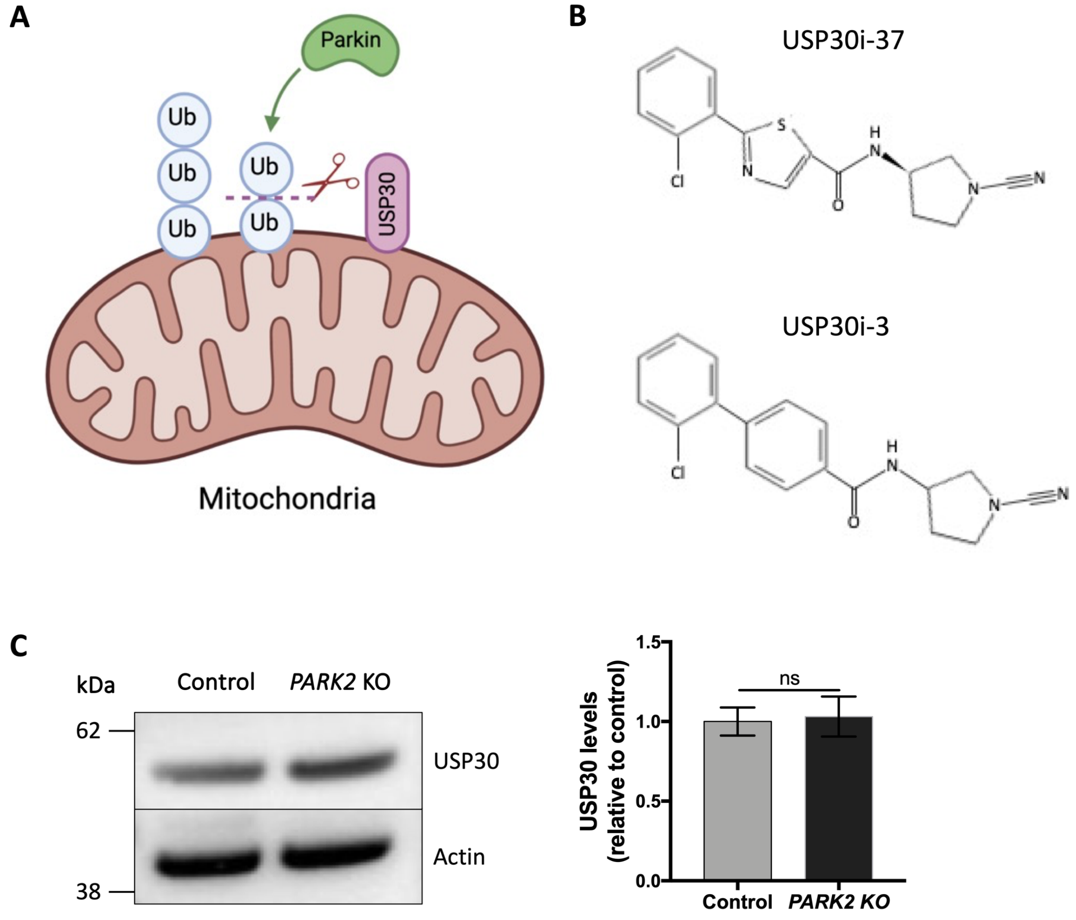

Figure S3: USP30 inhibition as a strategy to enhance mitophagy

A) USP30 opposes parkin-mediated ubiquitination. **B)** Chemical structure of USP30i-37 and USP30i-3. **C)** Western blotting and densitometric analysis demonstrating comparable levels of USP30 protein expression in *PARK2* KO and control neurons. Representative blots of two independent experiments.

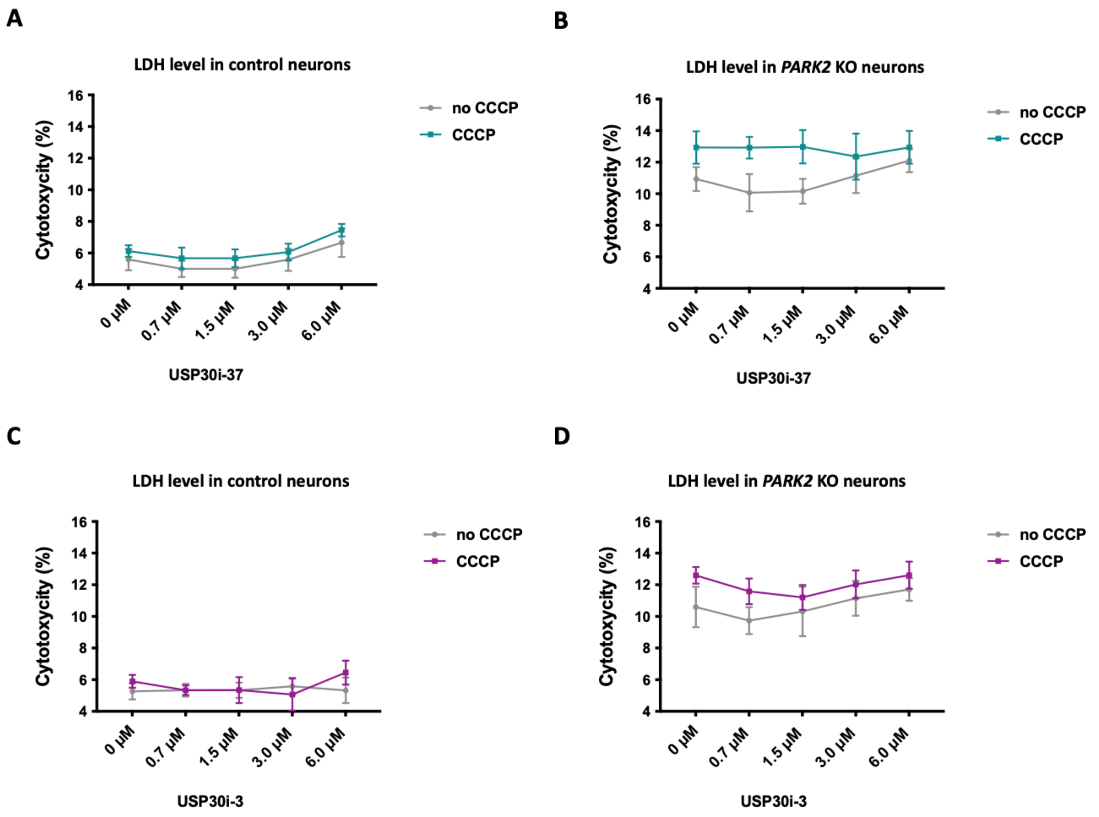

Figure S4: Lactate dehydrogenase (LDH) release did not reveal cytotoxic effects of USP30 inhibitors (+/-CCCP)

Differentiated neurons were treated with with 0 µM (untreated), 0.75 µM, 1.5 µM, 3 µM, 6 µM of USP30i-37 and USP30i-3 inhibitors added 4 h prior to CCCP (10 µM, 48 h). Necrotic cell death was assessed by LDH analysis of conditioned media from **A)** control neurons treated with USP30i-37, **B)** *PARK2* KO neurons treated with USP30i-37, **C)** control neurons treated with USP30i-3, and **D)** *PARK2* KO neurons treated with USP30i-3. No significant cytotoxicity was observed after treatment in either cell line, but there was a tendency to increase with increasing dose. Data presented as mean ± SEM, n=9-15 technical replicates, data from 3 independent differentiations.

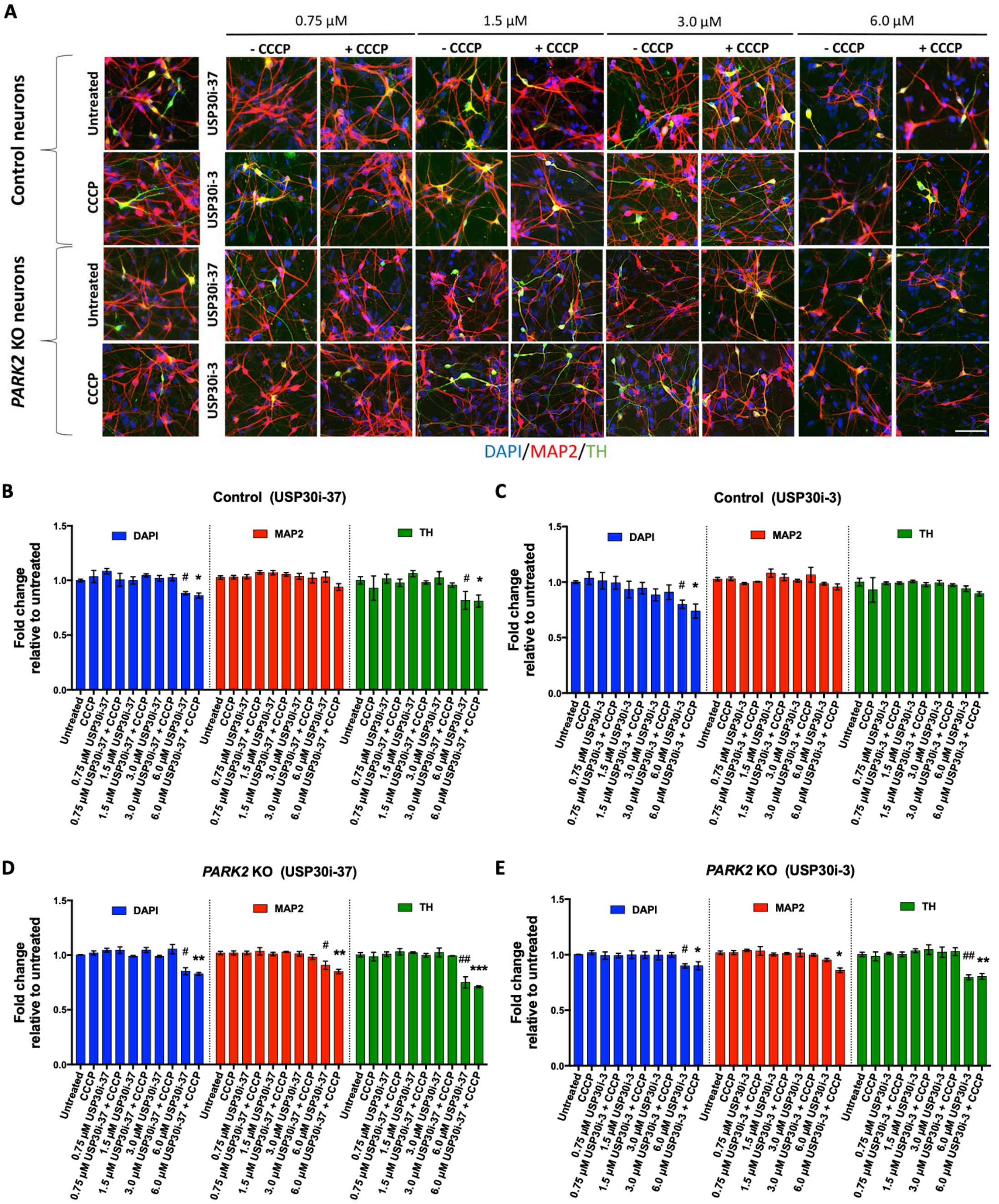

Figure S5: Immunofluorescence staining showed that 3 μM USP30 inhibitors did not affect the numbers of nuclei (DAPI), mature neurons (MAP2), or dopaminergic neurons (TH) (+/-CCCP)

Healthy control and *PARK2* KO neurons were treated with 0 μM (untreated), 0.75 μM, 1.5 μM, 3 μM, 6 μM of USP30i-37 and USP30i-3. Cells were fixed and stained for DAPI (blue), MAP2 (red), and TH (green). **A)** Representative immunoflorescence pictures of DAPI+, MAP2+, and TH+ cells in healthy control and *PARK2* KO iPSC-

derived neurons. Scale bars: 200 µm. **B-E)** Quantification of total cell count in **B, C)** healthy control and **D, E)** *PARK2* KO neurons. The toxicity of USP30 compounds (decreased cell number and compromised cell morphology) increased in both cell lines at the highest concentrations. Data presented as mean±SEM, n=9 technical replicates, data from 3 independent differentiations. Significant differences are indicated by $^{*,\#}p < 0.05$, $^{**,\#\#}p < 0.01$, $^{***}p < 0.001$, one-way ANOVA followed by Dunnett's post hoc test for multiple comparisons.

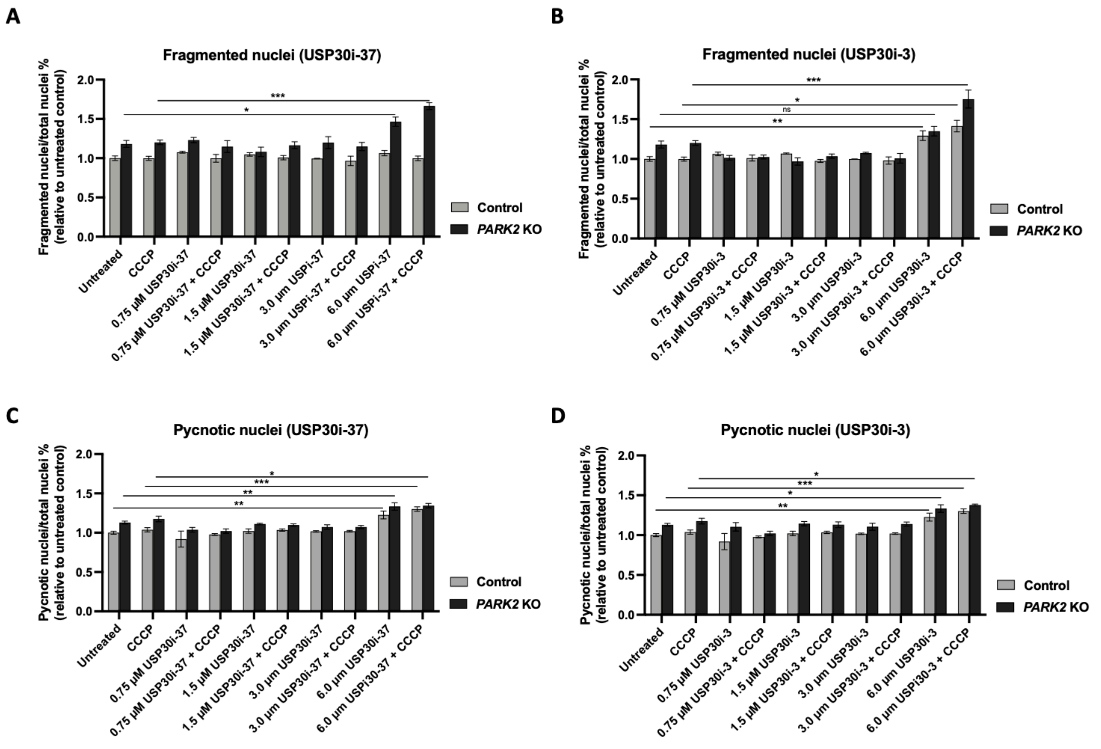

Figure S6: Morphological assessment of nuclei in control and *PARK2* KO neurons after treatment with USP30i-37 and USP30i-3.

Differentiated neurons were treated with with 0 μM (untreated), 0.75 μM, 1.5 μM, 3 μM, 6 μM of USP30i-37 and USP30i-3 inhibitors added 4 h prior to CCCP (10 μM, 48 h). Nucelar morphology was assessed by quantification of **(A-B)** fragmented nuclei in control and *PARK2* KO neurons treated with **A)** USP30i-37 and **B)** USP30i-3, and **(C-D)** pycnotic nuclei in control and *PARK2* KO neurons treated with **C)** USP30i-37 and **D)** USP30i-3. Data presented as mean±SEM, n=9 technical replicates, data from 3 independent differentiations. Significant differences are indicated by *'p < 0.05, **p < 0.01, ***p < 0.001, ns: non significant, one-way ANOVA followed by Dunnett's post hoc test for multiple comparisons.

Table S1. IC50 (nM) values for inhibition of the listed enzymes as determined at Ubiquigent

	USP30	USP21	UCHL1	USP15	AMSH-LP	USP2	USP6	USP25	USP45
**USP30i-3**	21	820	>3000	>3000	>3000	1600	60	2600	250
**USP30i-37**	48	430	>3000	>3000	>3000	1900	95	8700	400

Table S2. Enzymatic activity in the presence of 10 µM USP30i-3 as percentage of control (no inhibitor) as determined at Ubiquigent

Identity	Activity (%)	Identity	Activity (%)	Identity	Activity (%)
**USP1**	69.00	**USP25**	22.78	**OTU1**	82.19
**USP2**	25.60	**USP27x**	61.45	**OTUB2**	98.44
**USP4**	42.34	**USP28**	44.04	**OTUD1**	91.87
**USP5**	92.88	**USP30**	-0.08	**OTUD3**	88.69
**USP6**	0.21	**USP35**	69.81	**OTUD5**	95.24
**USP7**	82.29	**USP36**	57.79	**OTUD6A**	101.92
**USP8**	86.03	**USP45**	5.11	**OTUD6B**	80.95
**USP9x**	84.42	**CYLD**	92.11	**Cezanne**	102.95
**USP11**	58.48	**USP20**	73.06	**VCPIP**	104.01
**USP14**	99.18	**USP21**	11.38	**AMSH-LP**	95.41
**USP15**	57.50	**USP25**	22.78	**Ataxin3**	96.38
**USP16**	36.88	**UCHL1**	92.24	**Ataxin3L**	85.32
**USP19**	70.29	**UCHL3**	88.81	**JOSD1**	48.83
**USP20**	73.06	**UCHL5**	91.97	**JOSD2**	69.45
**USP21**	11.38	**BAP1**	91.49		
