# Supplementary material for: USP30 inhibition induces mitophagy and reduces oxidative stress in parkin-deficient human neurons
Source: Cell Death Dis. 2024 Jan 15;15(1):52. doi: 10.1038/s41419-024-06439-6 (PMC10789816; doi:10.1038/s41419-024-06439-6)
Supplement: Supplementary file 2 — Original Data File [file 41419_2024_6439_MOESM2_ESM.pdf]

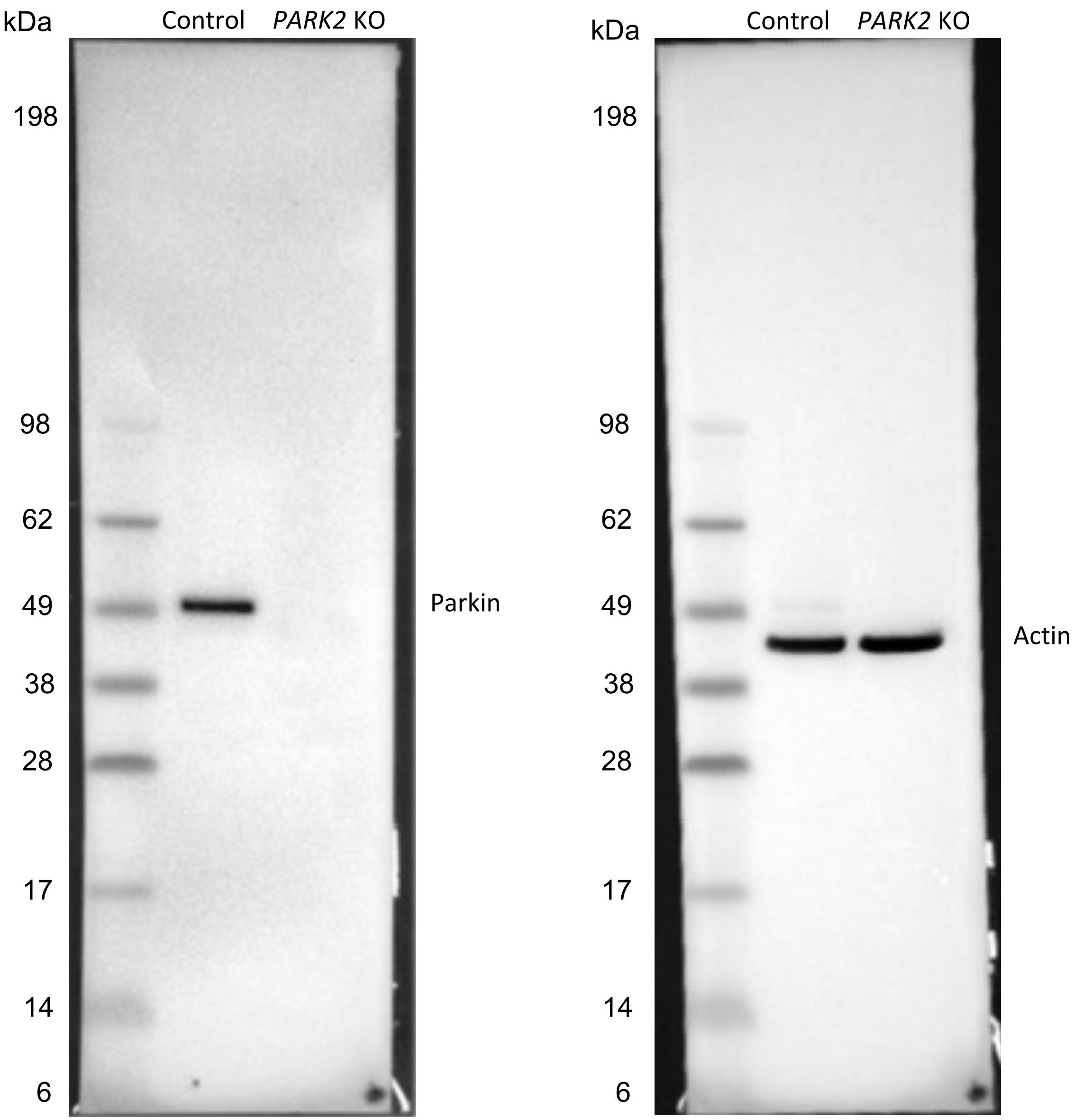

Full-size Western blots for Parkin and Actin.
Full-length blots referring to main Fig. 1B.

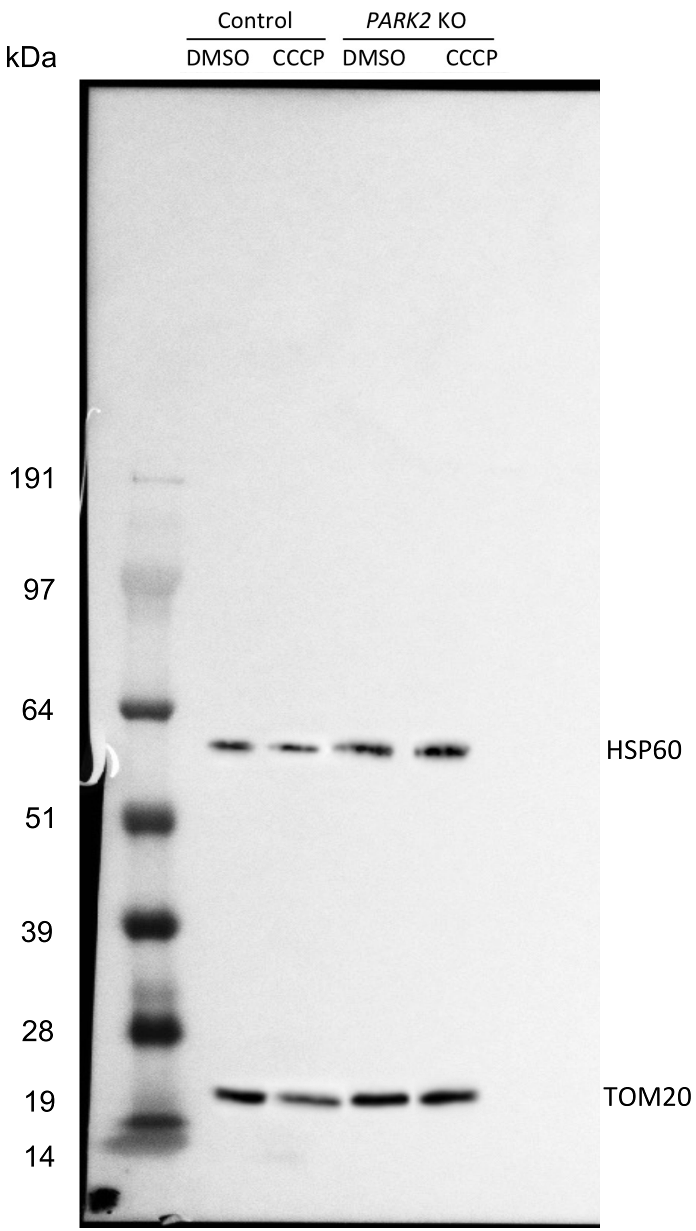

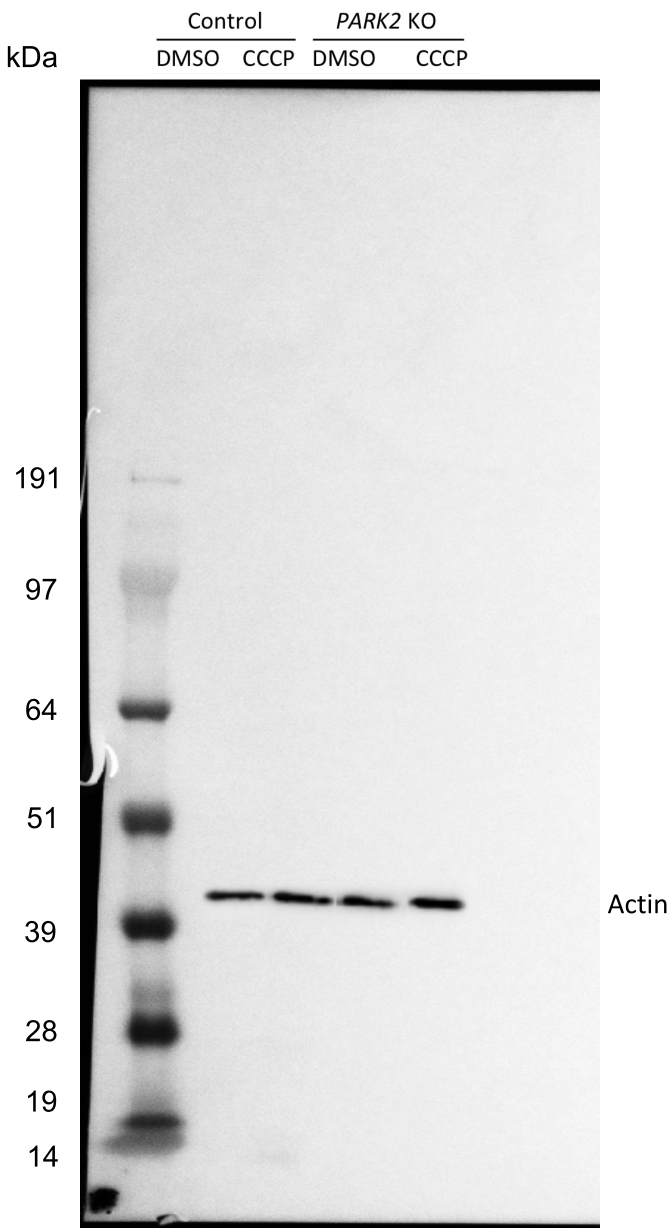

Full-size Western blots for TOM20, HSP60 and Actin.
Full-length blots referring to main Fig. 2C.

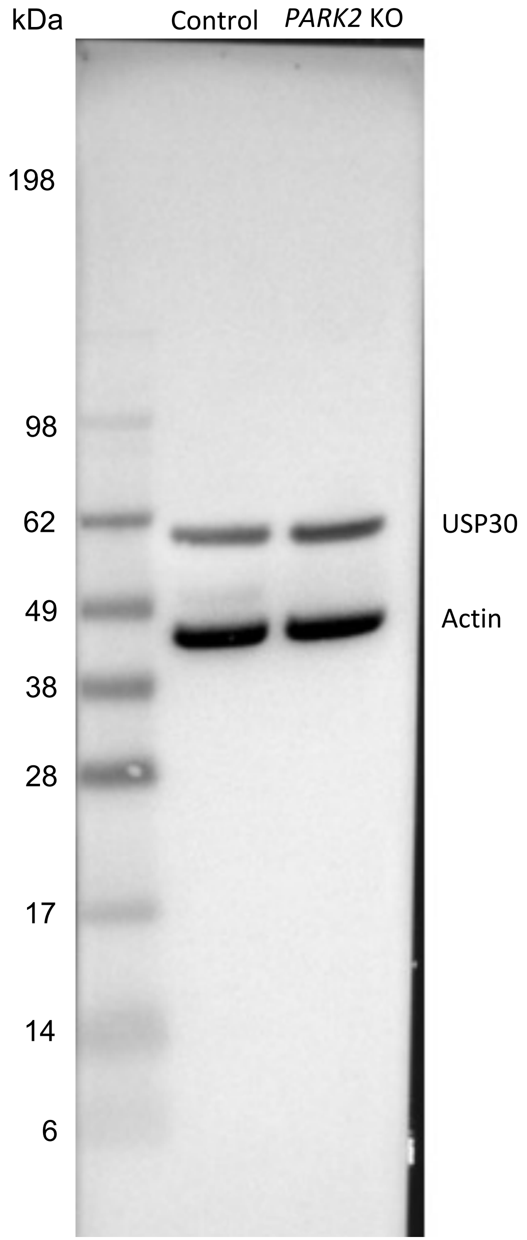

Full-size Western blots for USP30 and Actin.
Full-length blots referring to Supplementary Fig. S3C.
